# *Staphylococcus aureus* in Animals and Food: Methicillin Resistance, Prevalence and Population Structure. A Review in the African Continent

**DOI:** 10.3390/microorganisms4010012

**Published:** 2016-02-04

**Authors:** Carmen Lozano, Haythem Gharsa, Karim Ben Slama, Myriam Zarazaga, Carmen Torres

**Affiliations:** 1 Area of Biochemistry and Molecular Biology, University of La Rioja, Madre de Dios 53, Logroño 26006, Spain; carmencita_lf@hotmail.com (C.L.); myriam.zarazaga@unirioja.es (M.Z.); 2 Laboratoire des Microorganismes et Biomolécules Actives, Faculté de Sciences de Tunis, Université de Tunis El Manar, Tunis 2092, Tunisia; haythemgharsa@yahoo.fr (H.G.); Karim.BenSlama@fst.rnu.tn (K.B.S.); 3 Institut Supérieur des Sciences Biologiques Appliquées de Tunis, Université de Tunis El Manar, Tunis 1006, Tunisia

**Keywords:** MRSA, MSSA, CC398, CC130, CC133, Africa

## Abstract

The interest about *Staphylococcus aureus* (*S. aureus*) and methicillin resistant *S. aureus* (MRSA) in livestock, and domestic and wild animals has significantly increased. The spread of different clonal complexes related to livestock animals, mainly CC398, and the recent description of the new *mecC* gene, make it necessary to know more about the epidemiology and population structure of this microorganism all over the world. Nowadays, there are several descriptions about the presence of *S. aureus* and/or MRSA in different animal species (dogs, sheep, donkeys, bats, pigs, and monkeys), and in food of animal origin in African countries. In this continent, there is a high diversity of ethnicities, cultures or religions, as well as a high number of wild animal species and close contact between humans and animals, which can have a relevant impact in the epidemiology of this microorganism. This review shows that some clonal lineages associated with humans (CC1, CC15, CC72, CC80, CC101, and CC152) and animals (CC398, CC130 and CC133) are present in this continent in animal isolates, although the *mecC* gene has not been detected yet. However, available studies are limited to a few countries, very often with incomplete information, and many more studies are necessary to cover a larger number of African countries.

## 1. Introduction

*Staphylococcus aureus* (*S. aureus*) is a microorganism that is present as a commensal on the skin, the nose and mucous membranes of healthy humans and animals. However, it is also an opportunistic pathogen that can cause multiple infectious diseases of diverse severity. The epidemiology of this microorganism in animals has gained interest in the last years, not only because of their importance in veterinary medicine due to the increment of infectious processes caused by this pathogen (especially by methicillin-resistant *S. aureus* (MRSA) strains), but also because of the emergence of some clonal lineages associated with animals and their increasingly evidenced zoonotic potential. This is the case of the Sequence Type 398 (ST398), which has been identified as colonizer or infectious agent in pigs, cattle, horses, and poultry, as well as in people in contact with these animals (farmers, veterinarians, and slaughterhouse workers) [1,2,3,4,5,6]. Moreover, livestock associated (LA) MRSA infections have also been detected in relatives of farmers and some cases of MRSA of Clonal Complex 398 (CC398) have been identified in people without contact with animals [7]. These strains frequently exhibit multiresistance phenotypes. There are other clonal lineages (CC1, CC5, CC9, CC97, and CC130, among others) of LA-MRSA that are emerging, and whose importance is increasing in the last years. It should be pointed out that pets and wild animals can also act as reservoirs of MRSA strains, and play an important role in the epidemiology of this microorganism [5,8,9,10]. Recently, there has been growing interest not only in the study of MRSA strains but also of methicillin susceptible *S. aureus* (MSSA) strains, since these strains play an essential role in the evolution of different genetic lineages.

The number of studies focused on the antibiotic resistance problem in the African Continent has grown in last decade and they suggest that in this continent, as in other parts of the world, this problem is increasing; however, its real extent is currently unknown since surveillance of drug resistance is only carried out in a few countries [11]. The misuse of antibiotics due to poor control policies is promoting this resistance development [12]. Despite limited resources, during the last years in many of these countries, there are important efforts to establish good control measures to avoid this worrisome situation [13].

The study of *S. aureus* prevalence, antimicrobial resistance and clonal lineages in humans, animals and food in Africa has great relevance, taking into consideration the high diversity of ethnicities, cultures and religions that determine the lifestyle of African people. Most studies about MSSA and MRSA in the African continent are focused on human clinical isolates; nevertheless, the number of reviews focused on this topic is very scarce [14,15,16]. As would be expected, a higher diversity of clonal lineages is found among MSSA strains in comparison with MRSA strains, however, some clones (CC5 or CC8) have been found in methicillin resistant and susceptible strains [15]; a predominance of some clonal lineages (CC8 (ST239 and ST612), CC5, CC30, CC80, and CC88) has been identified in MRSA strains [15,16]. In many cases, CC88 is the dominant clonal lineage (24% to 83%) detected among MRSA strains in humans, and it has been named the “African clone” [15].

In this review, the objective is to report the situation of *S. aureus* in animals and food in Africa. The different African food habits highly influence the livestock industry of this continent. Moreover, there is a huge density and a high number of wild animal species that can be an important reservoir of this microorganism and of emerging antibiotic resistance mechanisms. These characteristics, and the close contact among humans, livestock, and domestic and wild animals, can have a relevant impact on the epidemiology of MSSA and MRSA. Therefore, it is essential to know what is happening, not only in strains from humans, but also in those of animal and food origin.

## 2. *S. aureus* in Animals in Africa

### 2.1. S. aureus Prevalence in Animals

**Figure 1:**
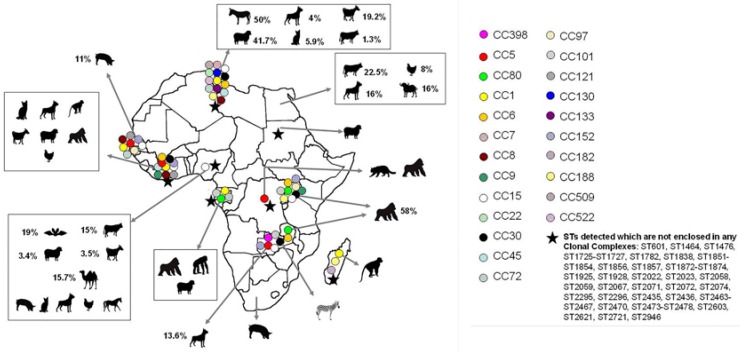
Animal species, clonal lineages and prevalence of MSSA identified in the Africa continent. Prevalence (%) is calculated considering the total number of samples of each animal species included in the different studies and is indicated when this estimation is possible with the data shown in each publication. Moreover, in those cases, the number of samples studied is also indicated (%/number of samples). Clonal complexes detected in more than one country are indicated as a triangle. The clonal complexes were determined by e-BURST when sequence types were indicated in the study and were presumptively assumed according to the *spa*-types when the sequences types were not indicated in the study.

**Figure 2:**
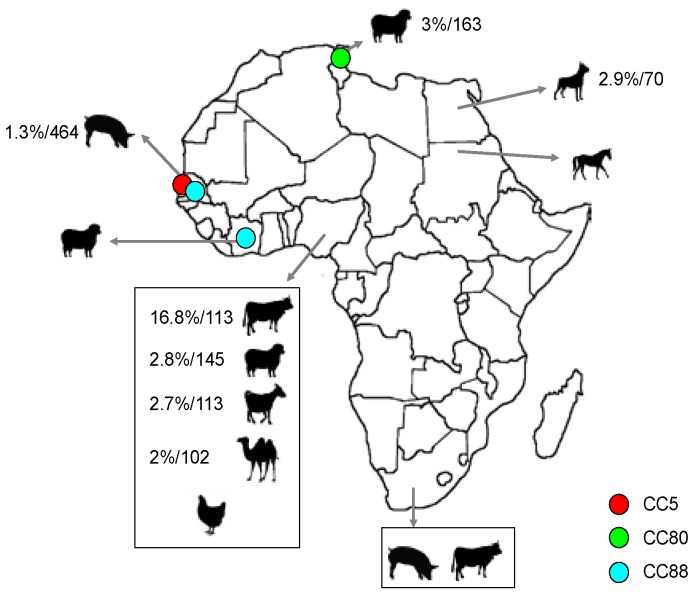
Animal species, clonal lineages and prevalence of MRSA identified in the Africa continent. Prevalence (%) is calculated considering the total number of samples of each animal species included in the different studies and is indicated when this estimation is possible with the data shown in each publication. Moreover, in those cases, the number of samples studied is also indicated (%/number of samples). The clonal complexes were determined by e-BURST when sequence types were indicated in the study and were presumptively assumed according to the *spa*-types when the sequences types were not indicated in the study.

Studies focused on the presence, prevalence and/or molecular typing of MSSA and MRSA strains from animals in Africa are rather limited and there is only information about certain countries (Table 1, Figure 1 and Figure 2) [17,18,19,20,21,22,23,24,25,26,27,28,29,30,31,32,33,34,35,36,37,38]. Until the moment when this review was performed, *S. aureus* strains had been reported in sick and healthy animals in 12 countries and MRSA strains had been only identified in seven of them (Côte d’Ivoire, Egypt, Nigeria, Senegal, South Africa, Sudan, and Tunisia). Most studies in animals have been performed in recent years, indicating an increased awareness of the role of animals in the evolution, epidemiology and dissemination of this microorganism.

The comparison of MSSA and MRSA prevalence data in the different studies carried out in the African continent is difficult due to the different methodologies that have been employed. In some studies, this prevalence is calculated taking into consideration the number of total samples, in others the number of each species included and in others the number of staphylococcal strains isolated. In Figure 1 and Figure 2, the prevalence was estimated considering the total number of samples of each species tested when these data were included in each publication. In this way, the MSSA prevalence identified in the different countries was highly variable (from 3% to 58%) (Figure 1). Some clonal lineages seem to be better adapted to some animal species, and *S. aureus* rate might be higher in these animals. This could be the case, for example, of CC130 and CC133 lineages, which are highly associated with ruminants, as detected in studies performed on other continents [39,40]. On the African continent, there might be other clonal lineages associated with certain animal species, but most of these clones are still unknown. There is a specific subclade (ST1874, ST2058, and ST2071) that seems to be related to monkeys according to one study carried out in sub-Saharan Africa [19]. Results shown in Table 1 can be influenced by the methodology of sampling, and thus, the oro-pharyngeal *S. aureus* colonization rate was higher (72%) than the rectal prevalence (8.7%) in lemurs [18]. Prevalence rates were also very different depending on the animal analyzed, being 11% in lemurs and 50%–80% in chimpanzees (vaginal samples in both cases). In addition, some studies were performed including healthy [34,35,36] and/or sick animals [21] (Table 1).

**Table 1:** Distribution and clonal lineages of *S. aureus* detected in animals in the African continent.

Country	Tested Animals	Animals from Which *S. aureus* Was Detected	Detection of MRSA	Animals from Which MRSA Was Detected	Sampling Date	Healthy/Sick	Samples	Lineages of MRSA	Lineages of MSSA ^b^	Reference
Côte d’Ivoire	Domestic and wild animals	Goats, cats, dogs, sheep, poultry, primates	yes	Sheep	2010–2013	Healthy	Nasal and pharyngeal	CC88	CC5, CC6, CC8, CC15, CC121, CC152, ST567, ST1472, ST2946, among others	[17]
	Primates	Chimpanzees	no	-	2007–2012	Healthy	Mucosal, feces, oral, genital, fruit wedges	-	CC1, CC45, ST601, ST1928, ST2603, ST2621	[18]
	Primates	Monkeys	no	-	-	Healthy	Nasal and fruit wedges	-	CC1, CC9, CC45, ST601, ST1782, ST1928, ST2023, ST2058, ST2059, ST2072, ST2603, ST2621	[19]
Democratic Republic of Congo	Domestic and wild animals	Civet, primates	no	-	2010–2013	Healthy	Nasal and pharyngeal	-	CC5, ST2473-ST2478, among others	[17]
Egypt	Dogs and cats	Dogs	yes	Dogs	-	Healthy and sick	Nasal, oral, ear, wound	HA-MRSA and CA-MRSA	-	[20]
	Cattle, dogs, buffaloes, poultry	Cattle, dogs, buffaloes, poultry	yes	No specified	-	Sick	Milk, wounds, abscesses, internal organs, urine and nasal	ND ^a^	-	[21]
Gabon	Primates	Gorillas, chimpanzees	no	-	2011	Healthy and sick	Nasal, oral, vaginal, rectal	-	CC72, CC101	[22]
	Primates	Monkeys, gorillas, chimpanzees	no	-	-	Healthy	Nasal and fruit wedges	-	CC1, CC80, ST1851-ST1854, ST1856, ST1857, ST1872 ST1874, ST1928, ST2022, ST2023, ST2067, ST2071, ST2074	[19]
	Domestic and wild animals	Sheep, primates	no	-	2010–2013	Healthy	Nasal and pharyngeal	-	CC101, CC80, ST1838, ST1851-ST1854, ST1857, ST1872-ST1874, ST1925, ST2022, ST2067, ST2071, ST2074, ST2295, ST2296, ST2721, among others	[17]
Madagascar	Primates	Lemurs	no	-	2007–2012	Healthy	Mucosal, feces, oral, genital, fruit wedges	-	CC1, CC182, CC188, ST2435, ST2436	[18]
Nigeria	Dogs, cats, chickens, pigs, horses, sheep, cattle, goats	Dogs, cats, chickens, pigs, horses, sheep, cattle, goats	-	-	-	Healthy and sick	Skin lesions, nasal, cloacal, milk	-	-	[23]
	Bats	Bats	no	-	2008–2010	Healthy	Fecal	-	CC15, ST1725-ST1727, ST2463-ST2467, ST2470	[24]
	Camels, sheep, cattle, goats	Camels, sheep, cattle, goats	yes	Camels, sheep, cattle, goats	2012	Healthy	Nasal and milk	ND	-	[25]
	Chickens	Chickens	no	-	-	Healthy	-	-	-	[26]
Senegal	Pigs	Pigs	yes	Pigs	2009–2011	Healthy	Nasal	CC5, CC88	CC1, CC5, CC8, CC15, CC72, CC97, CC121, CC152	[27]
South Africa	Pigs, cattle, goats, chickens	Pigs	yes	Pigs	-	Healthy	Nasal, mouth wash, ear	ND	-	[28]
	Chimpanzees	Chimpanzees	no	-	2007, 2010, 2011	Healthy	Nasal and oral	-	CC15, CC6, CC30, CC80, CC101	[29]
	Cattle and pigs	Cattle and pigs	yes	Cattle and pigs	-	Healthy	Rump, flank, brisket, neck	ND	-	[30]
Sudan	Sheep	Sheep	no	-	2007–2008	Sick	Abscesses	-	ST1464	[31]
	Sheep	Sheep	no	-	2003–2005	Sick	Pus samples	-	-	[32]
	Horse	Horse	yes	Horse	-	Sick	Lungs and peritoneum	ND	-	[33]
Tunisia	Sheep	Sheep	yes	Sheep	2010	Healthy	Nasal	CC80	CC8, CC130, CC522, ST1476, ST2076	[34]
	Donkeys	Donkeys	no	-	2010	Healthy	Nasal	-	CC1, CC6, CC7, CC15, CC22, CC72, CC133, CC522	[35]
	Cattle, goats, dogs, cats	Cattle, goats, dogs, cats	no	-	2010–2011	Healthy	Nasal	-	CC6, CC15, CC30, CC45, CC130, CC133, CC188, CC522	[36]
Uganda	Chimpanzees	Chimpanzees	no	-	2007, 2010, 2011	Healthy	Nasal and oral	-	CC15, CC6, CC30, CC80, CC101	[29]
	Primates	Chimpanzees	no	-	2007–2012	Healthy	Mucosal, feces, oral, genital, fruit wedges	-	CC6, CC9, CC15, CC30, CC80, CC152, CC188	[18]
Zambia	Zebra	Zebra	-		-	Sick	Tissue	-	-	[37]
	Chimpanzees	Chimpanzees	no	-	2007, 2010, 2011	Healthy	Nasal and oral	-	CC15, CC6, CC30, CC80, CC101	[29]
	Dogs and cats	Dogs	no	-	2012	Sick	Skin, ear, wound	-	CC398, CC5, CC15, CC152	[38]

^a^ ND, non-determined in the study. ^b^ The clonal complexes were determined by e-BURST when sequence types were indicated in the study and were presumptively assumed according to the *spa*-types when the sequences types were not indicated in the study. Sequence Types instead of Clonal Complexes were indicated when they were not enclosed in any Clonal Complexes.

In general, the MRSA colonization of animals detected in the African continent was very low (from 0% to 3%) [20,25,27,34], except for one study carried out in Nigeria [25], in which a colonization rate of 16.8% was observed in cattle samples (Figure 2). In countries on other continents, MRSA prevalence in healthy pets is usually lower than 1%, being between 9% and 20% in animals admitted to veterinary hospitals [41]. In one study performed in Egypt, MRSA isolates were identified in 2.9% of the analyzed dog samples and MRSA were not identified among the tested cat samples [20]. In livestock animals (especially in pigs), higher MRSA prevalence has been identified in European countries (4%–80%) [42]. In other farm animals, such as poultry, cattle and horses, the detected rates are normally lower than 13% [11,43,44]. On the African continent, MRSA has been found in variable rates in different livestock animals (cattle, sheep, pigs, goats, horses and camels) (Figure 2).

### 2.2. Population Structure of MSSA in Animals

As can be seen in Table 1 and Figure 1, a high diversity of clonal lineages has been identified among MSSA strains from animals in Africa. Twenty-three Clonal Complexes (CCs), 47 Sequence Types (STs) (which are not enclosed in any CC), and a many different *spa*-types were identified among these MSSA strains. Moreover, in these studies, numerous new STs [24,34,35,36,38] and *spa*-types [34,35] were detected. The most frequently found clonal lineages were CC1 and CC15, which were detected in the following countries: Côte d’Ivoire, Gabon, Madagascar, Nigeria, Senegal, Tunisia, Uganda and/or Zambia (Table 1 and Figure 1). In addition to CC1 and CC15, other clonal lineages (CC5, CC6, CC8, CC30, CC80, CC101, CC121, CC152, and CC188) were also identified in several African countries (Figure 1). Alternatively, some CCs were only observed in animals in one country. This is the case of CC398 in Zambia [38], CC130 and CC133 in Tunisia [34,35,36], and CC182 in Madagascar [18], among others.

Regarding clonal lineages associated with animals, there is only one description of MSSA CC398 in Africa, detected in the skin sample of one dog [38]. No other descriptions of MSSA or MRSA CC398 have been performed in other pets, in livestock animals or in wild animals in Africa. However, this clonal lineage has recently been identified in one MRSA clinical isolate in a hospital of Tunisia [45], and in MRSA and MSSA isolates from food samples, also in Tunisia, as will be detailed later [46]. Remarkably, other livestock associated CCs of high relevance have also been found in animals in Africa (Table 1 and Figure 1). MSSA CC130 strains were identified in Tunisia in sheep, goats, and one cow [34,36]. Monitoring of this lineage is very important since the new gene *mecC* has been identified mainly in CC130 strains in Europe [47,48]. However, this gene has not yet been found in any African country. CC133 is frequently found in ruminants [49,50,51] and this clonal lineage was identified in healthy donkeys in one study performed in Tunisia, and was the predominant CC found in 44% of the recovered isolates [35].

Other clonal lineages (CC1, CC5, CC8, CC9, CC30, CC97, and CC121) detected in animals in Africa have also been identified in livestock animals in other continents. CC1 has been previously found in pigs, cattle, poultry and horses in other studies [52,53,54], and identified in pigs, donkeys and non-human primates in Côte d’Ivoire, Gabon, Madagascar, Senegal, and Tunisia [17,18,19,27,35]. *S. aureus* strains belonging to CC5 are able to cause important infections in poultry [52]. Few studies about the presence of *S. aureus* in poultry have been performed in the Africa continent. Thus, MSSA strains have only been identified in poultry in Côte d’Ivoire, Egypt and Nigeria [17,21,23]. In one of these studies, molecular typing was performed and CC152 (and not CC5) was identified [16]. Although CC5 has not been found in poultry strains in Africa, this clone has been identified in MRSA and MSSA strains from other animal species (pigs, civets, dogs and goats) [17,27,38]. In Asian countries (China, Malaysia, and Thailand), the most common MRSA clone found in pigs is CC9 [55,56] and in Portugal CC30 (in addition to CC398) [57]. These CCs were identified in MSSA strains from animal species, except for pigs, in Côte d'Ivoire, Tunisia, Uganda, and/or Zambia [18,19,29,36]. However, it must be taken into consideration that the presence of this microorganism in pigs has only been studied for four works [23,27,28,30], and only in one of them the strains have been characterized [27]. In that study, the CCs identified in MSSA strains from pigs were CC1, CC5, CC72, CC97, CC121, CC15, CC152 and CC8 [27]. Regarding CC8, CC97 and CC121, these clonal lineages have been identified on other continents in cattle, horses, pigs, and rabbits [53,58,59,60,61].

### 2.3. Population Structure of MRSA in Animals

There are few studies in which MRSA strains have been identified in animals in Africa and only in three of them there is information about the ST or the CC detected [17,27,34] (Table 1 and Figure 2). The CCs identified were CC5 in pigs [27], CC80 in sheep [34] and CC88 in pigs and sheep [17,27]. CC5 and CC80 were also identified in MSSA strains in these and/or other studies [17,18,27,29,38]. Moreover, the three CCs found in MRSA of animals in Africa have been frequently detected among human clinical MRSA isolates in this continent [16,17]. MRSA isolates with the *mec*C gene have not yet been reported in Africa.

### 2.4. S. aureus Interspecies Transmission

MSSA and MRSA human-to-animal transmission has been suggested in some African studies [17,22,29]. Human related clonal lineages (CC15, CC72, CC80, CC101, and CC152) have been identified in MSSA strains from non-human primates, goats, sheep, poultry and pets [17,29]. Moreover, MRSA CC88 strains with the same *spa*-type (t189) were identified in humans and sheep in Côte d’Ivoire. In that study, samples were taken from domestic animals that lived in the same villages where the tested humans lived [17]. Another human-to-animal case transmission was identified in a sanctuary in Africa in which a veterinarian and a chimpanzee showed MSSA strains with the same *spa*-type t279 [29]. In addition, in the study of Nagel *et al.* [22], interspecies transmission of a widely spread human associated *S. aureus* CC72 strain (*spa*-type t148) was observed; this strain was found as colonizer agent in three gorillas and caused infection in one of them. Strains with the same *spa*-type were identified in chimpanzees in contact with the infected gorilla. These strains presented only one different band in Pulsed-Field Gel Electrophoresis (PFGE) compared with the strains obtained from gorillas [22].

On the other hand, in one study performed in Tunisia, nasal swabs of healthy people with different levels of interaction with animals were analyzed, and animal associated clonal lineages (CC30 and CC121) were found in some MSSA strains from people with frequent contact with animals [62]. In these cases, animal-to-human transmission might have happened.

## 3. *S. aureus* in Food in Africa

There are a high number of African studies focused on the microbiological analyses of food products (milk, meat, ready-to-eat, fish and eggs, among others). However, in most of them the main objective was usually to analyze the presence of different pathogens (among them, *S. aureus*), and to count CFU (Colony Forming Units) in order to determine the rate of contamination of the tested food [63,64,65,66,67,68]. In other studies, milk samples of sick animals were analyzed with the objective of detecting the presence of *S. aureus* as the mastitis-causing agent [69,70,71,72,73,74,75,76,77]. There were also a few papers in which the presence of *S. aureus* and/or MRSA was studied in food products from healthy animals. However, clonal lineages were determined only in a few of them [46,78].

### 3.1. MSSA Detection in Food Samples

MSSA strains have been identified in very diverse types of food in Africa in very different percentages (Table 2) [79,80,81,82,83,84,85,86,87,88,89,90,91,92,93,94,95,96,97,98,99,100,101,102,103,104,105,106]. The rates detected in raw meat, meat products and cooked meat have been from 3% to 81.8%. Cooked and uncooked samples were analyzed in one study carried out in Libya [91], and the prevalence was higher in raw chicken (29.6%), than in cooked meat (3.12%). In this case, it was demonstrated that the cooking process reduced the presence of this microorganism. However, the highest prevalence (81.8%) in meat samples was detected in one study performed in Cameroon in which cooked pork samples were analyzed [79]. In this case, human contamination during processing of these foods could be the most probable explanation. Some explanations of why this microorganism is present in food samples are given in the different publications. The fact that animals are kept in kitchens where food is prepared; direct contamination by the food handlers through coughing and sneezing; storage of food at high temperature; and/or some processed foods, which constitute a good culture medium for bacteria, are some of the possible reasons [88,97]. In addition, in the case of raw meat samples the source of contamination could also originate in the animal.

There are some methods such as molecular typing or determination of Immune Evasion Cluster (IEC) genes that could help us to know if the origin of *S. aureus* strains in meat samples might be human or animal [107,108]. However, clonal lineages were only determined in two studies [46,78]. In one of them, CC8, CC22, and CC398 were identified among chicken, sheep, and veal samples [46]. As previously noted, this was the first study in which CC398 has been found in food samples in the African continent [46]. In this study, twenty different *spa*-types were identified among MSSA strains. One of these *spa*-types (t1166) has been associated with CC133, and was detected in one strain isolated from a horse sample [46]. In another study carried out in Gabon, five MSSA strains were obtained from chicken samples [78]; three of them presented the *spa*-type t002 and belonged to ST5 (CC5), one showed the *spa*-type t386 and belonged to the singleton ST2622, and the remaining one had *spa*-type t591 and was non typeable by Multilocus Sequence Typing (MLST). Notably, the *spa*-type t002 was also identified in humans in Gabon [78]. It is important to mention that in this study, food samples were imported from industrialized countries (Brazil, Spain, USA, and Turkey), and it would be interesting to know if these clonal lineages are frequent in those countries. Until now, there is very scarce information about the *spa*-types t386 and t591. On the other hand, the *spa*-type t002 is widely spread all over the world [108].

Regarding milk samples from healthy animals, *S. aureus* prevalence was between 6.3% and 100% (Table 2). Most of the studies included raw milk samples from cattle or camels [83,85,104], although some of them also analyzed dairy products typical of the African continent, such as lben or jben [94]. One study carried out in Uganda analyzed the presence of different microorganisms in egg samples, and detected higher prevalence of *S. aureus* on the outer shell surfaces (18%) than inside the eggs (4%) [105]. Other types of food that have been analyzed include the following: beans, corn flour, doughnut, fish roll, salted fish, maize flour porridge, mangoes, meat pie, salad, pawpaw, and cassava [82,92,97,99,101] (Table 2).

### 3.2. MRSA Detection in Food Samples

**Figure 3:**
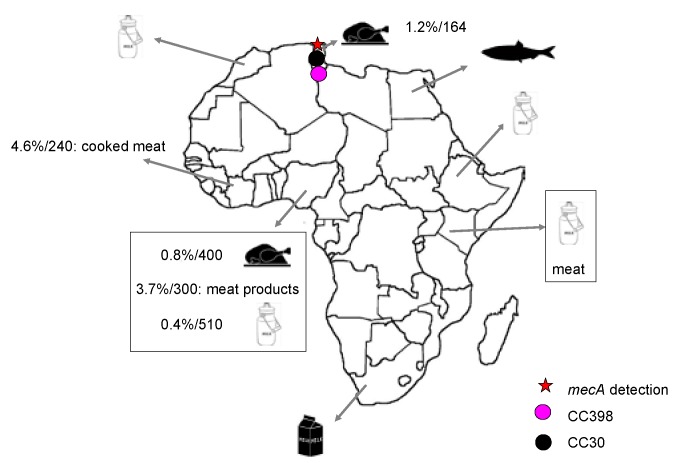
Food products, clonal lineages and prevalence of MRSA identified in the Africa continent. Prevalence (%) is calculated considering the total number of samples of each animal species included in the different studies and is indicated when this estimation is possible with the data shown in each publication. Moreover, in those cases, the number of samples studied is also indicated (%/number of samples). The clonal complexes were determined by e-BURST.

MRSA strains have been identified in meat, fish and milk samples from healthy animals in some studies performed in Africa (Table 2) (Figure 3). However, most of these MRSA strains have been identified by phenotypic methods, and the presence of the *mecA* gene was either not studied or not detected in many of them. MRSA strains have been found in raw meat, in meat products, and in cooked meat in Côte d’Ivoire, Nigeria, and Tunisia [26,46,80,98]. The presence of the *mecA* gene was analyzed in two of these four studies [46,98], and in only one of them this gene was found [46]. Molecular typing techniques were used in this last study [46], and two clonal lineages were identified (CC30 and CC398) in the two MRSA strains isolated from raw chicken samples.

MRSA prevalence identified in meat samples in the African studies was in the range 0.8%–4.6% [26,46,80,98]. Interestingly, the highest percentage was identified in a unique study in which MRSA strains were found in cooked meat samples [80]. Salted fish samples were analyzed in Egypt and methicillin resistance was observed in 12 of the 95 *S. aureus* strains tested (12.6%) [82]. In five studies [25,85,89,94,100], the prevalence was calculated regarding the total *S. aureus* strains isolated, and the obtained values were variable. In the study performed in South Africa, the prevalence was 81.2%–93.2% in milk samples from communal farms and 5.7%–7% in those from commercial farms [100]. The percentages obtained in the remaining studies were 60.3% in Ethiopia [85], 28.57% in Nigeria [25], 15% in Morocco [94], and 7.8% in Kenya [89]. The presence of the *mecA* gene was not studied in any of them, and data regarding the clonal lineages of these MRSA strains were also not available.

**Table 2:** Detection of *S. aureus* in food from healthy animals in the African continent.

Country	Samples	Number of Samples Studied	Date of Sampling	Raw/Cooked	Detection of MRSA ^a^	*S. aureus* Prevalence ^b^	Reference
Cameroon	Pork	11	-	Cooked	ND ^a^	81.8%	[79]
Côte d’Ivoire	Beef, chickens, pork	240	2010	Cooked	Yes	7.9%	[80]
Democratic Republic of Congo	Beef	-	-	Raw	ND	-	[81]
Egypt	Sausage, hamburger	60	-	Raw	ND	-	[65]
	Liver, meat	60	-	Cooked	ND	-	[63]
	Fish (sardine, feseikh, molouha)	60	-	Cooked	Yes	-	[82]
	Milk	150	-	Raw	ND	41.2%	[83]
	Goat (milk and meat)	100	-	Raw	ND	58% milk 18% goat meat	[84]
Ethiopia	Milk	-	2011–2012	Raw	yes	100%	[85]
	Meat samples	100	-	Raw	ND	21.2%	[86]
Gabon	Chicken	151	2011–2012	Raw	no	3.3%	[78]
Kenya	Milk	-	-	Raw and cooked	no	-	[87]
	Milk, minced meat	96	-	-	yes	-	[88]
	Milk	-	2001–2002	Raw	yes	-	[89]
Lesotho	Cattle, pigs, sheep	237	-	Raw	ND	5%	[90]
Libya	Chicken burger	120	-	Raw and cooked	ND	29.6% raw 3.12% cooked	[91]
Malawi	Home cooked food	132	-	Cooked	ND	61% (63% maize flour porridge, 51% fish, 75% vegetables, 69% beans, 38% others)	[92]
Morocco	Turkey	96	2011–2012	Raw	no	-	[93]
	Milk, lben, jben	-	2005–2006	Raw and cooked	yes	-	[94]
	Meat and beef offal	156	2002–2004	Raw	ND	16%	[95]
Namibia	Milk	15	1995–1996	Cooked	ND	-	[96]
Nigeria	Ready-to-eat food	168	-	Raw and cooked	ND	33.3% (57.1% salad, 19.1% meat pie, 14.3% fish roll, 9.6% doughnut)	[97]
	Milk	510	2012	Raw	yes	30.4%	[25]
	Suya, balangu, kilishi, dambunnama, raw beef	300	-	Raw and cooked	yes	9.7%	[98]
	Chicken	400	-	Raw	yes	-	[26]
	Ready to eat food (meat, fish, vegetable)	880	-	Raw and cooked	ND	62%	[99]
Somalia	Milk	-	-	Raw and cooked	no	-	[87]
South Africa	Milk	28	-	Raw	yes	100%	[100]
	Milk	156	1995–1996	Cooked	ND	-	[96]
	Poultry	-	-	Raw	ND	24.1%	[64]
	Street food vending (beef, chicken, salad, gravy)	132	-	Raw and cooked	ND	3%	[101]
Sudan	Sausage	40	-	Raw	ND	-	[68]
	Milk	320	-	Raw	ND	8.8%	[102]
	Milk	90	-	Raw	ND	-	[103]
Tanzania	Milk	128	2003	Raw	ND	6.3%	[104]
Tunisia	Chicken, horse, sheep, veal	164	2010–2011	Raw	yes	26.2%	[46]
Uganda	Eggs	171	-	Raw	ND	18% surface 4% inside	[105]
Zimbabwe	Milk	140	2009–2010	Raw and cooked	ND	-	[106]

^a^ ND: non determined (methicillin resistance was not tested). ^b^ Prevalence is calculated considering the total number of samples included in the different studies when this estimation is possible with the data shown in each publication.

## 4. Other Important Characteristics of *S. aureus* from Animals and Food in Africa

### 4.1. Antimicrobial Resistance of S. aureus

In some of the papers included in this review, in addition to methicillin resistance, antimicrobial resistance patterns to other agents were shown [17,18,19,20,21,22,24,25,28,29,30,31,34,35,36]. In general, MRSA isolates presented resistance to other non beta-lactam agents in addition to methicillin resistance, while MSSA isolates showed susceptibility to most of the antimicrobials tested. This situation is similar in MRSA and MSSA isolates from humans, animals and food in other parts of the world. In the studies in which MRSA isolates were obtained, resistance to tetracycline (5%–84%), erythromycin (1.7%–100%), clindamycin (9%–97%), trimethoprim-sulfametoxazole (1.9%–78%), tobramycin (0%–36%), ciprofloxacin (0%–42%) or vancomycin (9%–46%) were identified in different percentages [17,20,21,25,28,30,34,35,36,80,82]. Remarkably, in Nigeria all MRSA isolates obtained from camels, sheep and cattle showed resistance to mupirocin and fusidic acid and these antimicrobials are not routinely used in veterinary medicine in this zone [25]. Regarding MSSA isolates, in most of the studies, these isolates were susceptible to other non-beta-lactam antimicrobials. Only some of these isolates showed resistance to penicillin, tetracycline, erythromycin or clindamycin [17,18,19,22,24,25,29,31]. Nevertheless, in some cases, penicillin resistance was high among MSSA isolates [36,46,62], as occurs in other parts of the world; in the case of remote African regions, this phenotype is very rare, not only in animals but also in humans [29]. It must be taken into consideration that there are many factors that could be influencing the phenotypes detected. For example, it has been observed that MSSA isolates from chimpanzees in the wild were less resistant to penicillin, than isolates from chimpanzees living in captivity [18].

### 4.2. Virulence Determinants

Africa is considered endemic for Panton-Valentine-Leukocidin (PVL)-positive MSSA isolates [15,109]. Worryingly, this leukocidin has been identified in some MRSA from animals in Côte d’Ivoire, Gabon, Democratic Republic of Congo, Senegal and Tunisia [17,27,34], and in MSSA isolates in Côte d’Ivoire, Senegal and Tunisia [18,27,29,36]. In one study, PVL was significantly more frequent in isolates from chimpanzees than from humans (28% *vs.* 10%) [19]. According to these data, the possible role of animals as reservoir of this important virulence factor in this continent must be considered.

Other relevant virulence genes such as *tst*, *eta*, *etb* or *etd* have also been identified in animal isolates in Africa [18,23,27,34,35]. Moreover, the presence of genes encoding staphylococcal enterotoxins (SEs) responsible for food poisoning was studied in some articles. Some of these genes, such as *sea*, *seb*, *sei*, *seh* or *seg*, have been identified in isolates from different animal species in Africa [27,29,34,35]. Remarkably, these genes have also been found in isolates from food samples. In Egypt, SE genes were identified in 20.7% of raw goat milk samples and 11.1% of meat samples [84]. In Nigeria, 269 strains of 552 (48%) isolated from ready to eat food were enterotoxigenic, enterotoxin A being the most commonly found toxin [99]. However, in another study performed in Kenya, enterotoxin C was the most frequently produced type [88]; in this study, the highest percentage of enterotoxigenic strains was detected among chicken samples [88]. However, in one study performed in raw camel milk samples in Sudan, only three strains of 25 tested presented the enterotoxin C (the variant *sec2*) and the *egc* cluster [102].

## 5. Conclusions

The number of articles about the antibiotic resistance problem in African countries, and in particular about prevalence and clonal lineages of *S. aureus* strains in this continent, has increased in recent years. However, the available information is limited to a few countries, and is generally incomplete. Most of these studies are focused on clinical isolates, but there are some papers in which strains from various animal species (non-human primates, cows, pigs, donkeys, sheep, pets, bats, and camels) are analyzed. As in other parts of the world, animal MSSA strains present higher genetic diversity than MRSA strains. Clonal lineages associated with animals have been identified in several African countries, and the detection of MSSA CC398, CC130 and CC133 strains stand out. However, there is very scarce information about potential reservoirs and ways of dissemination of these clones in Africa. Relevantly, numerous new sequence types and *spa*-types have been identified in isolates of animals on this continent. Until now, the new *mecC* gene has not been detected in African countries, and further studies searching for its possible presence are required. On the other hand, there are several studies in which MSSA and MRSA strains have been found in food samples from healthy animals. However, in only two of them molecular typing of the *S. aureus* strains was performed. Therefore, the data in this regard are still insufficient. It is essential to know more about the current situation in these countries to assess the role of the food chain in the transmission of MRSA. Surveillance of MSSA and MRSA in humans, animals (pets, livestock and wild animals), and food in Africa can be a powerful tool for a better understanding of the epidemiology of this microorganism and for establishing appropriate control measures.
